# Supplementary material for: Knowledge, attitudes and practices relating to HIV self-testing following its introduction in the Bas-Sassandra region of Côte d’Ivoire: the case of the ATLAS project
Source: PLoS One. 2026 Jan 29;21(1):e0314947. doi: 10.1371/journal.pone.0314947 (PMC12854474; doi:10.1371/journal.pone.0314947)
Supplement: S1 Table — (DOCX) [file pone.0314947.s002.docx]

# S1 Table: Questions about HIV self-testing Knowledge, attitude and practice

| **Dimension** | **Variable names** | **Question** | **Possible answer** |
| --- | --- | --- | --- |
| Knowledge | Ever heard about HIVST | Have you ever heard of HIV self-testing as a method of HIV testing?  For your information, the HIV self-test allows a person to test themselves, most often in a private setting of their choice, by collecting their own saliva or blood (finger prick), then taking the test and interpreting the result themselves. An HIV self-test is different from a rapid HIV test which is done by someone else (often a health worker) who collects a drop of blood (finger prick) and performs the test for the person. | 0. No  1. Yes  98. Don't Know  99. Prefer not to say |
| Attitudes | Interested to use HIVST for themselves | If you were offered a free HIV self-test, how interested would you be: To test yourself for HIV? | 0. Not at all interested  1. Somewhat interested  2. Very interested  98. Don't Know  99. Prefer not to say |
|  | Interested to use HIVST for sexual partners | If you were offered a free HIV self-test, how interested would you be to give it to your regular partner so that he/she can test himself/herself? | 0. Not at all interested  1. Somewhat interested  2. Very interested  98. Don't Know  99. Prefer not to say |
| Practices | Ever used HIVST | Have you ever used a self-test to test yourself for HIV?  For your information, the HIV self-test allows a person to test themselves, often at home or in another private setting, by collecting their own saliva or blood (finger prick), then taking the test and interpreting the result themselves. An HIV self-test is different from a rapid HIV test which is done by someone else (often a health worker) who collects a drop of blood (finger prick) and performs the test for the person. | 0. No  1. Yes  98. Don't Know  99. Prefer not to say |
