# Supplementary material for: Knowledge, attitudes and practices relating to HIV self-testing following its introduction in the Bas-Sassandra region of Côte d’Ivoire: the case of the ATLAS project
Source: PLoS One. 2026 Jan 29;21(1):e0314947. doi: 10.1371/journal.pone.0314947 (PMC12854474; doi:10.1371/journal.pone.0314947)
Supplement: S2 Table — (DOCX) [file pone.0314947.s003.docx]

# S2 Table: Questions about HIV-related knowledge used to construct the knowledge score

| Questions | Right answer |
| --- | --- |
| Have you heard of HIV or a disease called AIDS? | Yes |
| In your opinion, is it possible for a healthy-looking person to be infected with HIV? | Yes |
| In your opinion, can people get HIV through mosquito bites? | No |
| In your opinion, can people get HIV through witchcraft or other supernatural means? | No |
| In your opinion, can people get HIV by sharing food with someone who has HIV? | No |
| In your opinion, can people get HIV by having sex with someone who has HIV? | Yes |
| In your opinion, can people get HIV by sharing a toothbrush with someone living with HIV? | No |
| In your opinion, can people get HIV by cutting themselves with a sharp object? | Yes |
| In your opinion, can people get HIV by injection? | Yes |
| In your opinion, can people get HIV through blood transfusions? | Yes |
| Can you reduce the risk of getting HIV by using a condom every time you have sex? | Yes |
| Can you reduce the risk of contracting HIV by having only one sexual partner who is not infected and who has no other sexual partners? | Yes |
| If a person has a partner who is circumcised, does that reduce their risk of getting HIV? | No |
| If an HIV-infected person is on antiretroviral therapy, can this prevent HIV transmission to another person? | Yes |
| If a person infected with HIV is on antiretroviral therapy, can it cure him/her completely? | No |
| If a person infected with HIV is on antiretroviral therapy, can they live a long and healthy life? | Yes |
