# Supplementary material for: Knowledge, attitudes and practices relating to HIV self-testing following its introduction in the Bas-Sassandra region of Côte d’Ivoire: the case of the ATLAS project
Source: PLoS One. 2026 Jan 29;21(1):e0314947. doi: 10.1371/journal.pone.0314947 (PMC12854474; doi:10.1371/journal.pone.0314947)
Supplement: S3 Table — (DOCX) [file pone.0314947.s004.docx]

# S3 Table: HIV-related attitude questions used to construct the negative attitude score

| Questions | Stigmatizing answer/negative attitude |
| --- | --- |
| Would you be willing to take care of an HIV-infected family member in your own household? | No |
| Would you buy fresh vegetables from a shopkeeper or vendor if you knew that person was infected with HIV? | No |
| Would you agree that a teacher who has HIV but is not sick should be allowed to continue teaching at school? | No |
| Would you agree that children living with HIV should be allowed to attend school with children who do not have HIV? | No |
| Would you be willing to have a romantic relationship with someone who has HIV? | No |
