# Supplementary material for: Knowledge, attitudes and practices relating to HIV self-testing following its introduction in the Bas-Sassandra region of Côte d’Ivoire: the case of the ATLAS project
Source: PLoS One. 2026 Jan 29;21(1):e0314947. doi: 10.1371/journal.pone.0314947 (PMC12854474; doi:10.1371/journal.pone.0314947)
Supplement: S4 Table — (DOCX) [file pone.0314947.s005.docx]

## S4 Table: Odds ratios from the multivariable logistic regression for KAP relating to HIVST among men aged 15-49 years in Côte d’Ivoire

|  | **Already heard about HIVST** | | | **Interested in using HIVST for themselves** | | | **Interested in using HIVST for sexual partners** | | | **Already used HIVST** | | |
| --- | --- | --- | --- | --- | --- | --- | --- | --- | --- | --- | --- | --- |
| **Characteristics** | **OR (95% CI)^2^** | **p-value** | **Adjusted GVIF^2,3^** | **OR (95% CI)^2^** | **p-value** | **Adjusted GVIF^2,3^** | **OR (95% CI)^2^** | **p-value** | **Adjusted GVIF^2,3^** | **OR (95% CI)^2^** | **p-value** | **Adjusted GVIF^2,3^** |
| **Age group** |  | <0.001 | 1.3 |  | 0.25 | 1.8 |  | 0.24 | 1.9 |  | <0.001 | 2.0 |
| 15-24 years old | — |  |  | — |  |  | — |  |  | — |  |  |
| 25-34 years old | 1.94 (1.33 to 2.81) |  |  | 1.18 (0.88 to 1.59) |  |  | 1.13 (0.80 to 1.58) |  |  | 3.10 (1.65 to 5.80) |  |  |
| 35-49 years old | 1.78 (1.26 to 2.53) |  |  | 0.90 (0.66 to 1.23) |  |  | 0.83 (0.62 to 1.12) |  |  | 1.83 (0.83 to 4.03) |  |  |
| **Level of education** |  | <0.001 | 1.3 |  | <0.001 | 1.3 |  | <0.001 | 1.4 |  | 0.27 | 2.1 |
| None | — |  |  | — |  |  | — |  |  | — |  |  |
| Primary | 1.50 (0.96 to 2.34) |  |  | 1.56 (1.14 to 2.13) |  |  | 1.51 (1.13 to 2.02) |  |  | 1.78 (0.59 to 5.35) |  |  |
| Secondary or higher | 2.53 (1.72 to 3.71) |  |  | 1.95 (1.59 to 2.39) |  |  | 1.97 (1.56 to 2.49) |  |  | 1.86 (0.85 to 4.08) |  |  |
| **Wealth index^1^** |  | 0.021 | 1.6 |  | 0.52 | 1.9 |  | 0.28 | 1.8 |  | 0.012 | 2.1 |
| Poor | — |  |  | — |  |  | — |  |  | — |  |  |
| Neither poor nor rich | 1.43 (0.83 to 2.47) |  |  | 1.18 (0.85 to 1.62) |  |  | 1.26 (0.94 to 1.69) |  |  | 2.49 (0.88 to 7.00) |  |  |
| Rich | 1.92 (1.19 to 3.10) |  |  | 1.00 (0.64 to 1.57) |  |  | 1.09 (0.68 to 1.75) |  |  | 4.20 (1.58 to 11.2) |  |  |
| **Number of sexual partners over the last 12 months** |  | 0.003 | 1.6 |  | 0.004 | 1.5 |  | <0.001 | 1.6 |  | 0.36 | 1.8 |
| 0 partner | — |  |  | — |  |  | — |  |  | — |  |  |
| 1 partner | 1.08 (0.65 to 1.80) |  |  | 1.65 (1.17 to 2.33) |  |  | 2.51 (1.67 to 3.76) |  |  | 1.25 (0.60 to 2.61) |  |  |
| 2 partners or more | 1.85 (1.12 to 3.05) |  |  | 1.51 (0.98 to 2.31) |  |  | 2.11 (1.29 to 3.44) |  |  | 1.88 (0.71 to 4.97) |  |  |
| **HIV knowledge** |  | 0.14 | 1.5 |  | <0.001 | 1.4 |  | <0.001 | 1.3 |  | 0.36 | 1.8 |
| Poor | 0.55 (0.30 to 1.02) |  |  | 0.56 (0.45 to 0.70) |  |  | 0.58 (0.45 to 0.74) |  |  | 0.35 (0.06 to 1.98) |  |  |
| Moderate | — |  |  | — |  |  | — |  |  | — |  |  |
| Good | 1.03 (0.79 to 1.36) |  |  | 1.33 (1.07 to 1.66) |  |  | 1.51 (1.16 to 1.98) |  |  | 1.20 (0.70 to 2.08) |  |  |
| **Negative attitude towards PLHIV** |  | 0.004 | 1.4 |  | <0.001 | 1.4 |  | <0.001 | 1.7 |  | 0.16 | 1.8 |
| High | — |  |  | — |  |  | — |  |  | — |  |  |
| Low | 1.61 (1.15 to 2.26) |  |  | 1.93 (1.53 to 2.43) |  |  | 1.83 (1.45 to 2.31) |  |  | 1.85 (0.77 to 4.47) |  |  |
| Exposure to the media |  | 0.014 | 1.9 |  | 0.44 | 1.7 |  | 0.56 | 1.8 |  | 0.23 | 1.7 |
| Low | — |  |  | — |  |  | — |  |  | — |  |  |
| High | 1.54 (1.08 to 2.19) |  |  | 1.09 (0.86 to 1.39) |  |  | 1.06 (0.86 to 1.33) |  |  | 1.47 (0.76 to 2.83) |  |  |
| **Department** |  | 0.020 | 1.6 |  | 0.22 | 1.5 |  | 0.65 | 1.4 |  | 0.17 | 1.5 |
| San-Pedro | 0.87 (0.43 to 1.77) |  |  | 0.98 (0.56 to 1.72) |  |  | 0.86 (0.49 to 1.51) |  |  | 1.09 (0.45 to 2.62) |  |  |
| Soubre | 0.75 (0.42 to 1.34) |  |  | 0.83 (0.47 to 1.46) |  |  | 0.79 (0.43 to 1.45) |  |  | 0.89 (0.39 to 2.07) |  |  |
| Tabou | 0.45 (0.25 to 0.81) |  |  | 1.17 (0.66 to 2.06) |  |  | 1.10 (0.53 to 2.27) |  |  | 0.26 (0.07 to 0.91) |  |  |
| Other | — |  |  | — |  |  | — |  |  | — |  |  |
| ^1^The wealth index was calculated using multiple correspondence analysis (MCA) from household asset variables | | | | | | | | | | | | |
| ^2^OR = Odds Ratio, CI = Confidence Interval, GVIF = Generalized Variance Inflation Factor | | | | | | | | | | | | |
| ^3^GVIF^[1/(2*df)]. The wealth index was calculated using multiple correspondence analysis (MCA) from household asset variables such as household ownership of certain consumer goods. | | | | | | | | | | | | |
