# Supplementary material for: Knowledge, attitudes and practices relating to HIV self-testing following its introduction in the Bas-Sassandra region of Côte d’Ivoire: the case of the ATLAS project
Source: PLoS One. 2026 Jan 29;21(1):e0314947. doi: 10.1371/journal.pone.0314947 (PMC12854474; doi:10.1371/journal.pone.0314947)
Supplement: S5 Table — (DOCX) [file pone.0314947.s006.docx]

## S5 Table: Odds ratios from the multivariable logistic regression for KAP relating to HIVST among women aged 15-49 years in Côte d’Ivoire

|  | **Already heard about HIVST** | | | **Interested in HIVST for themselves** | | | **Interested in HIVST for sexual partners** | | | **Already used HIVST** | | |
| --- | --- | --- | --- | --- | --- | --- | --- | --- | --- | --- | --- | --- |
| **Characteristics** | **OR (95% CI)^2^** | **p-value** | **Adjusted GVIF^2,3^** | **OR (95% CI)^2^** | **p-value** | **Adjusted GVIF^2,3^** | **OR (95% CI)^2^** | **p-value** | **Adjusted GVIF^2,3^** | **OR (95% CI)^2^** | **p-value** | **Adjusted GVIF^2,3^** |
| **Age group** |  | 0.27 | 1.5 |  | 0.93 | 1.3 |  | 0.97 | 1.2 |  | 0.39 | 1.8 |
| 15-24 years old | — |  |  | — |  |  | — |  |  | — |  |  |
| 25-34 years old | 1.24 (0.95 to 1.62) |  |  | 0.95 (0.72 to 1.25) |  |  | 1.04 (0.75 to 1.43) |  |  | 1.31 (0.74 to 2.33) |  |  |
| 35-49 years old | 1.17 (0.84 to 1.62) |  |  | 0.98 (0.74 to 1.29) |  |  | 1.01 (0.75 to 1.37) |  |  | 1.55 (0.78 to 3.10) |  |  |
| **Highest level of education** |  | 0.023 | 1.4 |  | <0.001 | 1.5 |  | <0.001 | 1.4 |  | 0.37 | 2.1 |
| None | — |  |  | — |  |  | — |  |  | — |  |  |
| Primary | 1.39 (0.94 to 2.06) |  |  | 2.34 (1.77 to 3.10) |  |  | 2.60 (1.84 to 3.66) |  |  | 1.05 (0.54 to 2.06) |  |  |
| Secondary or higher | 1.70 (1.12 to 2.58) |  |  | 2.64 (1.75 to 3.99) |  |  | 3.00 (2.02 to 4.45) |  |  | 0.69 (0.38 to 1.25) |  |  |
| **Wealth index^1^** |  | 0.002 | 1.7 |  | 0.34 | 1.4 |  | 0.35 | 1.3 |  | <0.001 | 1.5 |
| Poor | — |  |  | — |  |  | — |  |  | — |  |  |
| Neither poor nor rich | 0.95 (0.55 to 1.65) |  |  | 1.06 (0.76 to 1.48) |  |  | 1.18 (0.80 to 1.73) |  |  | 0.54 (0.15 to 1.90) |  |  |
| Rich | 1.95 (1.08 to 3.53) |  |  | 1.39 (0.88 to 2.19) |  |  | 1.37 (0.87 to 2.16) |  |  | 2.77 (1.25 to 6.13) |  |  |
| **Number of sexual partners over the last 12 months** |  | 0.040 | 1.4 |  | <0.001 | 1.3 |  | <0.001 | 1.2 |  | 0.82 | 1.5 |
| 0 partner | — |  |  | — |  |  | — |  |  | — |  |  |
| 1 partner | 1.23 (0.77 to 1.96) |  |  | 2.16 (1.50 to 3.09) |  |  | 2.66 (1.81 to 3.93) |  |  | 1.16 (0.47 to 2.86) |  |  |
| 2 partners or more | 2.09 (1.16 to 3.79) |  |  | 3.10 (1.65 to 5.83) |  |  | 3.99 (1.94 to 8.21) |  |  | 1.42 (0.45 to 4.50) |  |  |
| **HIV knowledge** |  | 0.14 | 1.5 |  | <0.001 | 1.4 |  | <0.001 | 1.3 |  | 0.081 | 1.4 |
| Poor | 0.60 (0.35 to 1.03) |  |  | 0.38 (0.30 to 0.48) |  |  | 0.42 (0.33 to 0.52) |  |  | 0.21 (0.05 to 0.91) |  |  |
| Moderate | — |  |  | — |  |  | — |  |  | — |  |  |
| Good | 1.10 (0.73 to 1.66) |  |  | 0.89 (0.65 to 1.23) |  |  | 0.96 (0.70 to 1.30) |  |  | 0.97 (0.49 to 1.92) |  |  |
| **Negative attitude towards PLHIV** |  | 0.25 | 1.4 |  | <0.001 | 1.3 |  | <0.001 | 1.2 |  | 0.16 | 1.9 |
| High | — |  |  | — |  |  | — |  |  | — |  |  |
| Low | 1.17 (0.88 to 1.56) |  |  | 1.96 (1.56 to 2.46) |  |  | 1.92 (1.50 to 2.46) |  |  | 1.62 (0.81 to 3.21) |  |  |
| **Exposure to the media** |  | 0.035 | 1.4 |  | 0.24 | 1.9 |  | 0.68 | 1.6 |  | 0.12 | 1.8 |
| Low | — |  |  | — |  |  | — |  |  | — |  |  |
| High | 1.54 (1.02 to 2.34) |  |  | 1.19 (0.88 to 1.61) |  |  | 1.05 (0.81 to 1.37) |  |  | 1.48 (0.88 to 2.47) |  |  |
| **District** |  | 0.001 | 1.2 |  | 0.56 | 1.4 |  | 0.24 | 1.3 |  | 0.52 | 1.4 |
| San-Pedro | 1.20 (0.67 to 2.15) |  |  | 0.85 (0.50 to 1.45) |  |  | 0.89 (0.55 to 1.43) |  |  | 1.16 (0.48 to 2.78) |  |  |
| Soubre | 1.09 (0.63 to 1.89) |  |  | 0.79 (0.56 to 1.12) |  |  | 0.70 (0.48 to 1.02) |  |  | 0.83 (0.38 to 1.80) |  |  |
| Tabou | 0.37 (0.18 to 0.78) |  |  | 0.98 (0.54 to 1.77) |  |  | 1.17 (0.54 to 2.51) |  |  | 0.54 (0.18 to 1.60) |  |  |
| Other | — |  |  | — |  |  | — |  |  | — |  |  |
| ^1^The wealth index was calculated using multiple correspondence analysis (MCA) from household asset variables | | | | | | | | | | | | |
| ^2^OR = Odds Ratio, CI = Confidence Interval, GVIF = Generalized Variance Inflation Factor | | | | | | | | | | | | |
| ^3^GVIF^[1/(2*df)]. The wealth index was calculated using multiple correspondence analysis (MCA) from household asset variables such as household ownership of certain consumer goods. | | | | | | | | | | | | |
