# Supplementary material for: Knowledge, attitudes and practices relating to HIV self-testing following its introduction in the Bas-Sassandra region of Côte d’Ivoire: the case of the ATLAS project
Source: PLoS One. 2026 Jan 29;21(1):e0314947. doi: 10.1371/journal.pone.0314947 (PMC12854474; doi:10.1371/journal.pone.0314947)
Supplement: Attachment — Submitted filename: PLOSE ONE.docx [file pone.0314947.s007.docx]

**Knowledge, attitudes and practices relating to HIV self-testing following its introduction in the Bas-Sassandra region of Côte d’Ivoire: the case of the ATLAS project**

Arlette Simo Fotso^1,2^, Christian KOUKOBO^2^, Romain Silhol^3^, Arsène Kouassi Kra^2^, Marie-Claude Boily^3^, Anthony Vautier^4^ and Joseph Larmarange^2,1^ on behalf of the ATLAS team

*1 Institut National des Études Démographiques (Ined), France*

*2 Centre Population et Développement (Ceped), Université Paris Cité, Institut de Recherche pour le Développement (IRD), Inserm, France*

*3 MRC Centre for Global Infectious Disease Analysis, School of Public Health, Imperial College London, United Kingdom*

*4 Solidarité Thérapeutique et Initiatives pour la Santé (Solthis), France*

*Corresponding author:* [*joseph.larmarange@ird.fr*](mailto:joseph.larmarange@ird.fr) *(JL) &* [*arlette.simo-fotso@ined.fr*](mailto:arlette.simo-fotso@ined.fr) *(ASF)*

# Abstract

**Background**

Awareness of HIV status is crucial for accessing HIV care and prevention but remains suboptimal in West Africa. The ATLAS initiative, launched in Côte d’Ivoire, Mali, and Senegal, addressed this gap by distributing approximately 380,000 HIV self-testing (HIVST) kits from 2019 to 2021, primarily to key populations and their social networks. This study assessed levels and correlates of Knowledge, Attitudes, and Practices (KAP) related to HIVST in the Bas-Sassandra region of Côte d’Ivoire following ATLAS’s introduction.

**Method**

A cross-sectional population-based survey was conducted in the Bas-Sassandra region in 2021 among individuals aged 15-49. Bivariate statistics and multivariable logistic regressions were used to assess KAP levels and the associated factors.

**Results**

Although few participants reported having heard about HIVST (11%) or having used it (3%), most of them reported that if it were freely available, they would be interested/very interested in using it for themselves (76%), as well as for their sexual partners (75%). Education and wealth were positively associated with knowledge and positive attitudes towards HIVST among both men and women, whereas age was positively correlated to knowledge and use of HIVST among men only. The number of sexual partners over the last 12 months was positively associated with knowledge of HIVST and willingness to use HIVST for themselves or their sexual partners among both sexes. We also found that high HIV-related knowledge and low levels of stigma were positively associated with positive attitudes towards HIVST, while exposure to the media appeared to be correlated to knowledge of HIVST.

**Conclusion**

The high level of positive attitudes towards HIVST calls for a scaling up of access to HIVST in the region. Specific attention to groups with the worst KAP, such as the less educated, the poor or groups that are highly stigmatised, could enhance the success of such initiatives.

# 1. Introduction

HIV testing is a central element in the strategy to end the HIV epidemic, as awareness of HIV status is the entry point to HIV care and prevention. In Western and Central Africa, nearly 5 million people are living with HIV, and in 2023, only 81% of them were aware of their HIV status (1). This is well below the UNAIDS 2025 target of reaching 95% of people living with HIV (PLHIV) who know their HIV status (1), thus highlighting the presence of obstacles linked to the classic testing strategies and the need to improve access to HIV screening services through innovative technologies.

HIV self-testing (HIVST) recommended by the WHO since 2016 as a complement to classic screening strategies, is a new medical tool that reduces barriers to access to screening (2), especially in contexts characterized by high levels of HIV-related discrimination and stigma (1,3). Several studies have shown that, for many users, HIVST promotes discretion and autonomy, reaches first-time testers, and is associated with an increase in testing frequency (4–12). HIVST is very well accepted, particularly by key populations and vulnerable groups, and reaches people living with HIV (13–17). In addition, recent studies have demonstrated the positive impact of HIVST distribution on HIV diagnosis, HIV treatment coverage, HIV incidence, and HIV-related mortality at population level (18,19).

In order to help fill the testing gap and build on the STAR project in Southern and Eastern Africa (20), the ATLAS programme (*AutoTest de dépistage du VIH: Libre d’Accéder à la connaissance de son Statut*) received funding from the global health initiative UNITAID, through the NGO *Solidarité Thérapeutique et Initiatives pour la Santé* (SOLTHIS) and the French National Research Institute for Sustainable Development (IRD), in partnership with national authorities and civil society organizations . The aim of the programme was to introduce, implement and scale up HIVST in West Africa. The ATLAS initiative has distributed around 380,000 HIVST kits through several distribution channels between 2019 and 2021 in Côte d’Ivoire, Mali and Senegal (21). More than half of these kits (~200,000 kits) were distributed in Côte d’Ivoire. Alongside the ATLAS programme, UNITAID has also funded MTV Shuga Babi, a two-season multimedia campaign, broadcast in Côte d’Ivoire in 2019-2021, targeting mainly young people aged 15-24, promoting messages consistent with national AIDS strategies and highlighting existing resources. Additional details about the two programmes are provided in the methods section.

With an HIV prevalence of 2.2% among adults in 2023, Côte d’Ivoire is one of the Western African countries most affected by the HIV epidemic. However, in this country only 82% of PLHIV knew their status in 2023 (22).

The Shuga Babi and ATLAS programmes conducted a joint, original, population-based, cross-sectional survey in the Bas-Sassandra District of Côte d’Ivoire to add new data to the limited existing empirical evidence on knowledge of, attitudes towards and practices relating to HIVST (KAP) in Africa (16,23). In this study, we aimed to use this survey to assess the levels and correlates of knowledge of, attitudes towards, and practices relating to HIV self-testing following the introduction of HIVST by the ATLAS project in Côte d’Ivoire. Identifying correlates of KAP could provide additional evidence-based tools for policymakers, which would enable them to implement actions to improve HIVST uptake and, ultimately, HIV status awareness.

# 2. Method

## 2.1. Data

We used data from a household survey that was carried out in the Bas-Sassandra district in Côte d’Ivoire: the Shuga Babi - ATLAS Household Survey (SBAHS).

### 2.1.1. Study setting

The Bas-Sassandra district is located in the southwest of Côte d’Ivoire and is one of the country's fourteen administrative districts. According to the most recent census in 2014, the district had a total population of 1,057,241 people aged 16 to 49, with 70% living in rural areas. It is one of the wealthiest districts in the country, with the San Pedro port activities and its cocoa and coffee production. These agricultural activities regularly attract a significant number of migrants and seasonal workers, who come to work on farms or in the sex trade. (24). The district is subdivided into 8 sub-districts or departments, 31 sub-prefectures (urban settings) and 533 localities (rural settings).

### 2.1.2. Description of the ATLAS programme

The ATLAS programme distributed 74,785 HIVST kits in the Bas-Sassandra district over the years 2019-2021. More precisely, 42,997, 23,287, and 8,501 kits were distributed in the Soubré, San Pédro, and Tabou health sub-districts of the district respectively. The ATLAS programme mainly relies on two modes of distribution of HIVST kits. The first is the primary distribution strategy, where peer educators issued kits to the primary contacts for their personal use. The second is a secondary distribution, where primary contacts are issued with additional testing kits to redistribute to their sexual and social network – peers, partners and clients. The ATLAS activities are organized through eight different delivery channels: five facility-based approaches for the delivery of HIVST kits through public or community-based healthcare facilities, and three community-based approaches involving outreach activities targeting female sex workers (FSW), men who have sex with men (MSM), and people who use drugs (PWUD) (21). Approximately 90% of HIV self-testing (HIVST) kits were distributed to key populations. During the implementation period, HIVST kits were not available in pharmacies, and there was no other free distribution programme comparable to ATLAS in the district. The only exception was the United States President's Emergency Plan for AIDS Relief (PEPFAR), which distributed a relatively small number of HIVST kits — around 15,000 — over the same period in the district.

### 2.1.3. The MTV Shuga Babi Campaign

Alongside the ATLAS programme, the MTV Shuga Babi, a two-season multimedia campaign, was broadcast in Côte d’Ivoire over the years 2019-2021, targeting mainly young people aged 15-24. The first season of MTV Shuga Babi campaign combined issues of HIV transmission and prevention messages addressed to young people through entertaining stories tailored for them. The central component of MTV Shuga Babi broadcasts in 2019 was a television (TV) drama series linked to complementary media initiatives to ensure that people with little access to television were reached. Using the same approach, MTV implemented a second season, which was broadcast in 2021. The second season had one episode focusing on the existence and the benefits of HIVST, showing the administration of an HIVST kit on-screen, highlighting the importance of follow-up testing and treatment after a reactive HIVST test and portraying preventive behaviour and repeat testing for those testing negative. The aims of the campaign were simultaneously to raise awareness and to create a demand for HIVST and other prevention strategies among young people.

### 2.1.4. Data collection

The SBAHS survey was conducted between November 20^th^ and December 31^st^, 2021, about 24 months after the start of the ATLAS project activities (distribution of HIV self-tests), and 6 months after the television broadcast of the second season of the MTV series Shuga Babi. This was a joint survey of the ATLAS and MTV Shuga Babi programmes, aiming, on the basis of the interviewees’ reports, to assess the primary and secondary outcomes in the evaluations of the two initiatives. For the ATLAS programme, the survey aimed to quantify baseline HIV testing in the 12 months prior to the survey, HIVST awareness and uptake and links to care as primary and secondary outcomes.

SBAHS was a cross-sectional population-based survey designed to be representative of individuals aged 15 to 49 living in the study area. It used a three-stage stratified sampling approach. The district was divided into four areas, three corresponding to the three health sub-districts or departments where the ATLAS activities were concentrated (Soubré, San Pédro, Tabou) and the last one corresponding to the other health department. Each area was further divided according to urban / rural setting, resulting in 8 strata. In order to capture sufficient data in the three ATLAS departments for statistical analyses, the first three strata were over-represented, accounting for two-thirds of the total sample. Similarly, the urban setting was over-represented (about 50% of the sample compared to 30% in the general population) to enable the sample sizes in each setting (urban/rural) to be equal.

Firstly, enumeration areas (EA) were selected for each stratum with a probability proportional to size, resulting in a total of 60 EAs. Second, 60 households per EA were selected using simple random sampling. Third, all *de facto* household members eligible (aged 15-49 years) were invited to participate. Those who gave their written informed consent – or had written informed assent from parents/guardians for minors — were interviewed. Face-to-face interviews with a trained interviewer were conducted in French or in one of the main local languages, which are English, Godie, Fanti, Malinke, Kroumen, Bakoue, Baoule, Bete and Neo, using a structured questionnaire loaded on tablets. Ethics approvals were obtained from the Côte d’Ivoire Ministry of Health (N/ref: 051-21/MSHP/CNESVS-km), the London School of Hygiene and Tropical Medicine (LSHTM) (LSHTM Ethics Ref: 26258), and the World Health Organization (WHO) (ERC.0003596) ethics committees in June, July and September 2021 respectively. The survey was implemented in collaboration with the *École Nationale Supérieure de Statistique et d'Économie Appliquée in Côte d’Ivoire* (ENSEA).

## 2.2. Outcome variables

We defined four dichotomous dependent variables related to knowledge, attitudes, and practices relating to HIV self-testing. The first dependent variable measured individuals’ knowledge of HIVST, assessing whether the respondent “had already heard of HIV self-testing”. The second and third dependent variables concerned the respondents’ interest in using HIVST for themselves or for their partners if it was freely available. These were binary variables indicating whether the person was interested or not. These variables reflected the respondents’ attitudes towards HIVST. The fourth variable reflecting practices asked individuals whether they had ever used HIVST.

## 2.3. Independent variables

We grouped the independent variables used in this analysis into five sets.

**Socio-demographic and economic variables** included sex (men, women), the age group (15-24, 25-34, 35-49 years), the level of education (no formal education, primary, secondary or tertiary education), whether or not currently in a relationship, and the wealth index tercile (poor, neither poor nor rich, rich) derived from the wealth index. The index was calculated using multiple correspondence analysis (MCA) using household asset variables such as household ownership of certain consumer goods (TV, radio, etc.) and housing characteristics (availability of electricity, building materials, type of water supply, type of toilet, and fuel used for cooking).

**Behavioural variables** included the number of sexual partners in the last 12 months (0, 1, 2 or more) and an indicator relating to who made healthcare decisions for the respondent.

**The HIV-related knowledge and stigma** set included two variables. The categorical knowledge variable was based on a series of 16 questions about HIV knowledge (see supplement 1). The answers to this question were categorized into three levels: poor, moderate, and good, corresponding to fewer than 10, 10-12, and 13 or more correct answers respectively. The level of stigma was classified as high if the respondent gave a stigmatizing response to at least two of the five questions on HIV-related stigma (see Supplement 2), and low otherwise.

**Exposure to the media** was measured by a binary media exposure variable (low, high) based on the frequency of exposure to radio, television, and Internet access. Exposure was high if the individual was exposed to at least two media per week and low otherwise.

**Contextual variables** were also used as potential correlates, such as the place of residence (urban, rural) and the department (Soubré, San Pédro, Tabou and others).

## 2.4. Methodology

We first performed univariate and bivariate analyses to assess levels and factors associated with Knowledge, Attitudes, and Practices (KAP). Univariate analyses involved describing the characteristics of the sample. A cross-tabulation using the Chi² test with Rao & Scott’s second-order correction was applied to the bivariate analysis to examine the association between the outcome and independent variables, identifying significant associations at the 5% significance level.

To check whether the associations observed in bivariate analysis remained after controlling for other covariates, a quasi-binomial multivariable logistic regression was performed. Regressions were conducted separately for men and women to assess the KAP correlates in each group. The results are presented as adjusted Odds Ratios (aORs) with 95%-confidence intervals (CI). The latter accounts for model-robust standard errors, adjusting for data clustering. The adjusted Generalized Variance Inflation Factor (GVIF) (25) was used to ensure that there were no serious multicollinearity issues in the multivariate regression. It is important to note that some variables were excluded from all regressions for the multivariate analysis because they were a source of multicollinearity. Analyses were performed with R software (version 4.2.1) using the packages survey, gtsummary and ggstats. Unless otherwise specified, all analyses reported in this study were weighted and took the survey design into account.

# 3. Results

## 3.1. Participant characteristics

A total of 6,271 people (3,203 men and 3,068 women) were interviewed and completed the questionnaire (Table 1). A large proportion were in a relationship (77%), had no formal education (46%), and were aged 15-24 (36%). Most participants were living in rural areas (62%) and in the department of San Pedro (35%). Most reported that decisions about their own health were taken mainly by a third party (53%), and that they had had only one sexual partner over the 12 months preceding the survey (69%). Half of the population had an average knowledge of HIV, and 63% presented a low level of stigma towards PLHIV. Only 39% of the population had high exposure to the media.

With regard to KAP relating to HIVST, although very few participants reported having heard about HIVST (10% of the women and 11% of the men), most of them showed a positive attitude towards HIVST and reported that they would be interested/very interested in using it for themselves (73% of the women and 78% of the men), as well as for their sexual partners (72% of the women and 78% of the men), if it was freely available. About 3% of both women and men reported that they had already used a HIVST in the past.

Table 1: Characteristics of the study population by sex

|  | **Women** | **Men** | **Overall** |
| --- | --- | --- | --- |
| **Characteristics** | **(n=3068)1** | **(n=3203)1** | **(n=6271)1** |
| **Socio-demographic and -economic factors** |  |  |  |
| **Age group** |  |  |  |
| 15-24 years old | 38.4% (1,259) | 34.6% (1,192) | 36.4% (2,451) |
| 25-34 years old | 33.0% (953) | 29.7% (913) | 31.4% (1,866) |
| 35-49 years old | 28.6% (856) | 35.7% (1,098) | 32.2% (1,954) |
| **Level of education** |  |  |  |
| None | 55.8% (1,629) | 37.4% (1,205) | 46.4% (2,834) |
| Primary | 21.5% (637) | 24.3% (714) | 22.9% (1,351) |
| Secondary or higher | 22.7% (802) | 38.3% (1,284) | 30.6% (2,086) |
| **Wealth index** |  |  |  |
| Poor | 35.6% (1,029) | 36.2% (1,114) | 35.9% (2,143) |
| Neither poor nor rich | 30.0% (884) | 31.8% (959) | 30.9% (1,843) |
| Rich | 34.4% (1,155) | 32.0% (1,130) | 33.2% (2,285) |
| **Currently in a relationship** |  |  |  |
| No | 17.0% (575) | 29.3% (1,025) | 23.3% (1,600) |
| Yes | 83.0% (2,493) | 70.7% (2,178) | 76.7% (4,671) |
| **Behavioural factors** |  |  |  |
| **Number of sexual partners over the last 12 months** |  |  |  |
| 0 partner | 14.7% (447) | 18.2% (632) | 16.5% (1,079) |
| 1 partner | 81.1% (2,468) | 57.4% (1,804) | 69.0% (4,272) |
| 2 partners or more | 4.2% (145) | 24.3% (758) | 14.4% (903) |
| **Usually,** **who makes the decisions about your own health care?** |  |  |  |
| Myself | 11.4% (384) | 66.9% (2,080) | 39.7% (2,464) |
| A third party | 79.6% (2,430) | 28.4% (973) | 53.5% (3,403) |
| Myself and a third party | 8.9% (254) | 4.7% (150) | 6.8% (404) |
| **HIV-related knowledge and stigma** |  |  |  |
| **HIV knowledge** |  |  |  |
| Poor | 26.3% (816) | 18.6% (603) | 22.4% (1,419) |
| Moderate | 49.5% (1,496) | 47.0% (1,505) | 48.2% (3,001) |
| Good | 24.2% (756) | 34.3% (1,095) | 29.4% (1,851) |
| **Stigmatization towards PLHIV** |  |  |  |
| High | 39.5% (1,201) | 33.8% (1,110) | 36.6% (2,311) |
| Low | 60.5% (1,867) | 66.2% (2,093) | 63.4% (3,960) |
| **Exposure to the medias and contextual factors** |  |  |  |
| **Exposure to the media** |  |  |  |
| Low | 71.0% (2,124) | 51.3% (1,560) | 61.0% (3,684) |
| High | 29.0% (932) | 48.7% (1,635) | 39.0% (2,567) |
| **Place of residence** |  |  |  |
| Rural | 59.9% (1,453) | 63.4% (1,608) | 61.7% (3,061) |
| Urban | 40.1% (1,615) | 36.6% (1,595) | 38.3% (3,210) |
| **Department** |  |  |  |
| San-Pedro | 35.0% (843) | 34.3% (838) | 34.6% (1,681) |
| Soubre | 23.8% (879) | 24.3% (908) | 24.1% (1,787) |
| Tabou | 7.9% (367) | 10.2% (469) | 9.1% (836) |
| Other | 33.2% (979) | 31.2% (988) | 32.2% (1,967) |
| **Outcome variables** |  |  |  |
| **Already heard about HIVST** |  |  |  |
| No | 89.9% (2,762) | 88.9% (2,863) | 89.4% (5,625) |
| Yes | 10.1% (305) | 11.1% (338) | 10.6% (643) |
| **Interest in using HIVST for themselves** |  |  |  |
| Not interested | 27.3% (781) | 21.6% (679) | 24.4% (1,460) |
| Interested / very interested | 72.7% (2,255) | 78.4% (2,507) | 75.6% (4,762) |
| **Interest in using HIVST for sexual partners** |  |  |  |
| Not interested | 28.2% (806) | 22.3% (703) | 25.2% (1,509) |
| Interested / very interested | 71.8% (2,216) | 77.7% (2,472) | 74.8% (4,688) |
| **Already used HIVST** |  |  |  |
| No | 97.4% (2,988) | 97.4% (3,130) | 97.4% (6,118) |
| Yes | 2.6% (75) | 2.6% (69) | 2.6% (144) |
| 1% (n (non-weighted)). The wealth index was calculated using multiple correspondence analysis (MCA) from household asset variables. | | | |

## 3.2. Factors associated with HIVST knowledge

A bivariate analysis of the association between KAP and the independent variables is presented in Table 2. The first columns show that knowledge of HIVST was significantly and positively associated with age (p =0.035), education (p <0.001), wealth (p <0.001) and being in a relationship (p =0.009). Knowledge of HIVST was also significantly higher among people whose decision regarding their own health was taken by themselves and by a third party (p =0.008), for those with two or more sexual partners (p<0.001), high HIV-related knowledge (p <0.001), low HIV-related stigma (p <0.001), and high exposure to the media (p <0.001).

Table 2: Bivariate analysis of factors associated with KAP relating to HIVST among individuals aged 15-49 years in Côte d’Ivoire

|  | **Already heard about HIVST** | | **Interested in using HIVST for themselves** | | **Interested in using HIVST for sexual partners** | | **Already used HIVST** | |
| --- | --- | --- | --- | --- | --- | --- | --- | --- |
| **Characteristics** | **% (n)^1^** | **p-value^2^** | **% (n)1** | **p-value^2^** | **% (n)^1^** | **p-value^2^** | **% (n)1** | **p-value^2^** |
| **Sex** |  | 0.28 |  | 0.002 |  | <0.001 |  | 0.92 |
| Woman | 10.1% (305) |  | 27.3% (781) |  | 28.2% (806) |  | 2.6% (75) |  |
| Man | 11.1% (338) |  | 21.6% (679) |  | 22.3% (703) |  | 2.6% (69) |  |
| **Age group** |  | 0.035 |  | 0.17 |  | 0.029 |  | 0.002 |
| 15-24 years old | 9.3% (233) |  | 25.3% (577) |  | 26.8% (623) |  | 1.7% (42) |  |
| 25-34 years old | 12.3% (214) |  | 22.6% (413) |  | 22.7% (404) |  | 3.6% (59) |  |
| 35-49 years old | 10.4% (196) |  | 25.1% (470) |  | 25.8% (482) |  | 2.7% (43) |  |
| **Level of education** |  | <0.001 |  | <0.001 |  | <0.001 |  | 0.031 |
| None | 6.1% (168) |  | 33.9% (912) |  | 34.9% (941) |  | 1.7% (41) |  |
| Primary | 10.1% (132) |  | 18.6% (244) |  | 19.3% (255) |  | 2.9% (32) |  |
| Secondary or higher | 17.7% (343) |  | 14.4% (304) |  | 15.0% (313) |  | 3.8% (71) |  |
| **Wealth index** |  | <0.001 |  | 0.001 |  | 0.003 |  | <0.001 |
| Poor | 6.0% (138) |  | 29.7% (631) |  | 31.1% (658) |  | 1.1% (21) |  |
| Neither poor nor rich | 8.2% (136) |  | 24.4% (428) |  | 24.4% (425) |  | 1.5% (19) |  |
| Rich | 17.8% (369) |  | 18.6% (401) |  | 19.6% (426) |  | 5.3% (104) |  |
| **Currently in a relationship** |  | 0.009 |  | 0.093 |  | <0.001 |  | <0.001 |
| No | 8.6% (139) |  | 26.6% (406) |  | 31.6% (477) |  | 1.4% (23) |  |
| Yes | 11.2% (504) |  | 23.7% (1,054) |  | 23.3% (1,032) |  | 3.0% (121) |  |
| **Number of sexual partners over the last 12 months** |  | <0.001 |  | <0.001 |  | <0.001 |  | 0.003 |
| 0 partner | 7.6% (79) |  | 33.4% (345) |  | 39.1% (404) |  | 1.5% (13) |  |
| 1 partner | 9.7% (416) |  | 23.5% (942) |  | 23.4% (936) |  | 2.5% (100) |  |
| 2 partners or more | 17.9% (145) |  | 18.0% (166) |  | 18.1% (163) |  | 4.3% (29) |  |
| **The person who generally makes the decisions about their own health care** |  | 0.008 |  | <0.001 |  | <0.001 |  | 0.004 |
| Themselves | 12.3% (289) |  | 20.8% (515) |  | 21.1% (517) |  | 2.9% (65) |  |
| A third party | 8.7% (304) |  | 27.1% (848) |  | 28.3% (894) |  | 2.0% (63) |  |
| Themselves and a third party | 15.9% (50) |  | 24.0% (97) |  | 25.0% (98) |  | 5.8% (16) |  |
| **HIV knowledge** |  | <0.001 |  | <0.001 |  | <0.001 |  | <0.001 |
| Poor | 4.9% (78) |  | 42.8% (560) |  | 43.4% (570) |  | 0.6% (9) |  |
| Moderate | 10.5% (301) |  | 21.5% (634) |  | 22.9% (671) |  | 2.8% (70) |  |
| Good | 15.0% (264) |  | 15.4% (266) |  | 15.4% (268) |  | 3.9% (65) |  |
| **Stigmatization towards PLHIV** |  | <0.001 |  | <0.001 |  | <0.001 |  | <0.001 |
| High | 6.3% (137) |  | 36.0% (808) |  | 36.9% (835) |  | 1.2% (24) |  |
| Low | 13.1% (506) |  | 17.8% (652) |  | 18.5% (674) |  | 3.5% (120) |  |
| **Exposure to the media** |  | <0.001 |  | <0.001 |  | <0.001 |  | <0.001 |
| Low | 7.3% (261) |  | 28.2% (1,000) |  | 28.8% (1,023) |  | 1.7% (57) |  |
| High | 15.8% (382) |  | 18.4% (454) |  | 19.5% (479) |  | 4.1% (87) |  |
| **Place of residence** |  | 0.089 |  | 0.15 |  | 0.22 |  | 0.29 |
| Rural | 8.9% (250) |  | 26.1% (779) |  | 26.7% (796) |  | 2.2% (53) |  |
| Urban | 13.3% (393) |  | 21.7% (681) |  | 22.7% (713) |  | 3.4% (91) |  |
| **Department** |  | 0.10 |  | 0.72 |  | 0.64 |  | 0.086 |
| San-Pedro | 13.4% (210) |  | 23.1% (413) |  | 24.0% (430) |  | 3.9% (53) |  |
| Soubre | 10.1% (197) |  | 27.0% (450) |  | 28.8% (476) |  | 2.3% (43) |  |
| Tabou | 4.4% (44) |  | 23.6% (180) |  | 22.6% (171) |  | 0.8% (11) |  |
| Other | 9.7% (192) |  | 24.0% (417) |  | 24.5% (432) |  | 2.0% (37) |  |
| 1% (n (non-weighted)) | | | | | | | | |
| 2 Pearson's X^2: Rao & Scott adjustment. The wealth index was calculated using multiple correspondence analysis (MCA) from household asset variables. | | | | | | | | |

The multivariable analysis presented in Figure 1 and Table A1 for men and in Figure 2 and Table A2 for women helps to apprehend the persistence of these associations after controlling for other variables. Overall, the tables show that after testing the model and removing the variables that were a source of multicollinearity – the variables *currently in a relationship*, *who makes the decisions about your own health care* and *place of residence* — in the final model there was no strong multicollinearity issue in the regressions, (aGVIF is <$\surd5\approx2.23)$ (26,27).

**[Figure 1: Odds ratios from the logistic regression for KAP relating to HIVST among men aged 15-49 years in Côte d’Ivoire ]**

**[Figure 2: Odds ratios from the multivariable logistic regression for KAP relating to HIVST among women aged 15-49 years in Côte d’Ivoire]**

Among the men, age, education and wealth proved to be positively associated with knowledge of HIVST. Men aged 35-49 years (aOR: 1.78 [95%CI: 1.26 to 2.53]) and 25-34 years (aOR: 1.94 [1.33 to 2.81]), those with secondary or higher education (aOR =2.53 [95% CI: 1.72 to 3.71], those from the highest wealth tercile (aOR:1.92 [95%CI: 1.19 to 3.10]), those with two sexual partners or more (aOR:1.95 [95%CI: 1.12 to 3.05]), those with low levels of stigmatization towards PLHIV (aOR:1.61 [95%CI: 1.15 to 2.26]), and those with high exposure to the media (aOR:1.54 [95%CI: 1.08 to 2.19]) had higher odds of having heard of HIVST compared to reference groups of those aged 15-24 years, with no education, belonging to the poorest wealth tercile, with no sexual partner, those expressing high stigmatization toward PLHIV and low media exposure respectively. People from the Tabou had lower odds of HIVST knowledge (aOR:0.45 [95%CI: 0.25 to 0.81]) than those living in other departments. Similar patterns of association were found among women except for age and the stigmatization variables, which appeared non-significantly correlated to HIVST knowledge.

## 3.3. Factors associated with attitudes towards HIVST

The participants’ interest in using HIVST for their sexual partners if freely available in the future (column 3 in table 2) appeared to be significantly associated with all independent variables, except the department and place of residence. In addition to the last two variables, the participants' interest in using HIVST for themselves in the future was not significantly associated with age group or being in a relationship.

Multivariate analysis (columns 2 in table A2 and figure 2) showed that women with secondary or higher education (aOR:2.64 [95%CI: 1.75 to 3.99]), primary education (aOR:2.34 [95%CI: 1.77 to 3.10]), with one sexual partner (aOR:2.16 [95%CI: 1.50 to 3.09]), with two or more sexual partners (aOR:3.10 [95%CI: 1.65 to 5.83]) and low stigmatization towards PLHIV (aOR:1.96 [95%CI: 1.56 to 2.46]) had higher odds of being interested in using HIVST for themselves, while those with low HIV-related knowledge (aOR:0.38 [95%CI: 0.30 to 0.48]) had lower odds. On the other hand, compared to the reference groups, women who had secondary or higher education (aOR:3.00 [95%CI: 2.02 to 4.45]), primary education (aOR:2.60 [95%CI: 1.84 to 3.66]), one partner (aOR:2.66 [95%CI: 1.81 to 3.93]), 2 or more sexual partners (aOR:3.99 [95%CI: 1.94 to 8.21]) and low stigmatization towards PLHIV (aOR:1.92 [95%CI: 1.50 to 2.46]) were more likely to be interested in using HIVST for sexual partners. They were less likely to be interested when they had, a poor level of HIV knowledge (aOR:0.42 [95%CI: 0.33 to 0.52]) compared to those with moderate knowledge (see column 3 in table A2).

A similar pattern of association was found among men for both interest in using HIVST for themselves and for their sexual partners (see columns 2 and 3 in table A1 and figure 1).

## 3.4. Factors associated with HIVST practice

Bivariate analyses showed that the use of HIVST was positively associated with age (p=0002), with the age group 25-34 having the highest proportion of HIVST use (4%). HIVST use was also positively associated with education (p=0.031), wealth (p<0.001), the number of sexual partners (p=0.003), the level of HIV-related knowledge (p<0.001), low stigmatization towards PLHIV (p<0.001), exposure to the media (p<0.001) and being in a relationship (p<0.001) (see the fourth column in table 2).

After controlling for other factors, in multivariable analysis, it appeared that men aged 25-34 (aOR:3.10 [95%CI: 1.65 to 5.80]) and from the wealthiest tercile (aOR:4.20 [95%CI: 1.58 to 11.2]) had a higher odd of already used HIVST than those aged 15-25 and from the poorest tercile. Among the women, only wealth was significantly associated with the use of HIVST, with women from the wealthiest tercile having odds of already used HIVST, 2.77 times greater than those from the poorest tercile [95% CI: 1.25 to 6.13]

# 4. Discussion and Conclusion

This study aimed to assess levels and correlates of knowledge of, attitudes towards, and practices relating to HIV self-testing in the general population after its introduction by the ATLAS programme in Côte d’Ivoire. It drew on a representative survey carried out in the Bas-Sassandra region at the end of 2021.

The results showed that about 24 months after the introduction of HIVST in the region via the ATLAS programme, knowledge of HIVST remained relatively low overall (11%). The actual use of HIVST was even lower (3%). Although this proportion seems low, it is higher than results found by Terefe *et al.* among women in 21 Sub-Saharan African countries between 2015 and 2022. They found that prevalence of knowledge, and use of HIVST among women was about 2.17% (95% CI: 2.12-2.23) (23) – although the measures used by the authors were not really comparable to ours, as they did not enable a distinction between knowledge and use.

The levels of HIVST knowledge that we found in the study population seems surprisingly high, given the scale of the ATLAS programme. Indeed, the ATLAS initiatives were of rather modest proportions given the size of the population in the region – 74,785 HIVST kits were distributed over a three-year period on a territory of about 1,057,241 individuals aged 16-49 years. In addition, the programme activities were circumscribed, targeting key and vulnerable populations (and their partners), and especially those remote from the healthcare system but contributing substantially to the HIV spread because of the prevention gaps. About 90% of the ATLAS HIVST kits were distributed to key populations, accounting for less than 3% of the total population – about 1.4 % of women for FSW and 1.3% of men for MSM in 2019 (19).

The restricted targeted population of the ATLAS programme was in line with national recommendations regarding priority populations, on the basis of prevalence rates. As a result, in order to avoid any stigmatization, since only a few specific groups had access to HIVST, the strategies for creating demand were designed to reach the targeted populations exclusively. Consequently, no communication on HIVST targeted the general population, which ultimately had an impact on the level of knowledge and use of HIVST observed in this study.

We found very high levels of positive attitudes towards HIVST, with three quarters of the respondents expressing willingness to use it for themselves and their sexual partners, if freely available. These results are in line with those found in ATLAS qualitative research among sex workers in West Africa, which found that they were willing to use and/or reuse HIVST and distribute it to regular clients and partners (16). Similar proportions were found in Kenya and Rwanda, among young adults living in informal urban settlements and among male clinic attendees respectively (28,29). In line with our study, several surveys have shown a higher acceptance of HIVST among men than among women (10), probably because the former are usually more remote from the healthcare system than women who are likely to receive antenatal care. Confidentiality, empowerment, responsibility and saving of time and financial resources related to eventual transportation costs to HIV testing facilities are some of the reasons explaining the overall positive attitudes towards HIVST (14,30–33).

Multivariate analysis showed that exposure to the media appeared to be correlated with HIVST knowledge. This could be explained by the MTV Shuga multimedia campaign, which included an episode in its second season promoting HIVST. However, this result should be interpreted cautiously, because although we cannot rule out the possibility that other non-targeted age groups may have been exposed to it, the MTV-Shuga multimedia campaign was designed to target mostly the youngest people (aged 15-24 years), and our results showed that knowledge, attitudes and practices in this age group were no better than in the older age group. In fact, the opposite is true. Exposure to the media (TV, Internet and the radio) could be a proxy for better general culture.

The place of residence did not appear to be strongly correlated with KAP relating to HIVST despite differences in ATLAS activities intensity. This could be partly due to the fact that the ATLAS activities were fairly modest and targeted, as previously explained. On the other hand, because of population mobility across departments, information and HIVST kits could also have circulated across departmental boundaries, making it difficult to observe any difference between departments initially targeted by the ATLAS programme activities and those receiving almost no distribution of kits.

Regarding the socio-demographic and socio-economic factors, education was positively associated with knowledge and positive attitudes towards HIVST for both men and women. Wealth was positively associated with knowledge and practices concerning HIVST in the two groups. This corroborates previous evidence in the literature on Sub-Saharan African countries and China (23,34–36). It is usually explained by the fact that education and wealth are associated with knowledge of HIV status, use of healthcare services, and health-seeking behaviours. Age appeared to be positively correlated with knowledge and use of HIVST among the men only. Previous research has found that age is positively associated with HIVST knowledge and use for both genders (23,28). This could be explained by life experiences or personal levels of risk-taking, but also by higher costs of going to a healthcare facility to undergo a standard HIV test for older adults.

Behavioural factors were also associated with KAP relating to HIVST, with a positive association found between the number of sexual partners over the last 12 months and the participants’ knowledge of HIVST and willingness to use it for themselves or for their sexual partners among both men and women. The effect was even stronger for those with two or more sexual partners, corroborating studies that have shown that individuals with risky sexual behaviours are more likely to know and accept HIVST (29). Contrasted results were found in other studies, although they focused on different, younger populations than the one in this study (28,37).

We also found that, if HIV-related knowledge and stigma did not appear to be associated with knowledge and practice of HIVST, especially among the women, it was an important correlate of attitudes towards HIVST for both men and women. This could be explained by the fact that a good knowledge of HIV generally implies awareness of the benefits of knowing one’s own HIV status, while low stigma reduces their fear of receiving a positive HIV test result, making them more willing to use HIVST if available to them. In fact, the literature suggests that HIV-related stigma is an important barrier to testing (38). Previous studies have also found a significant association between having negative attitudes towards HIVST and high HIV-related stigma, though this association did not show in multivariable analysis (39).

This work presents some limitations. Firstly, because of budget limitations, the survey was restricted to, and is representative of, only one region in Côte d’Ivoire, making it difficult to extrapolate the results to other regions or countries. However, there is no serious reason to think that our results would be completely different from those that might be observed in other Ivorian regions. Secondly, the sample size did not allow for a specific analysis of key populations or the ATLAS targeted groups. However, some qualitative studies and a telephone survey targeting these groups have already been conducted as part of the ATLAS project, and they have shown that HIVST is very well accepted, particularly by key populations, and does indeed reach people living with HIV (13,16,40). The cross-sectional nature of the survey makes it difficult to establish a cause-and-effect relationship, so future studies could adopt a study design that addresses this issue.

Nevertheless, the current study contributes to the literature in different ways. Firstly, it enabled an assessment, at population level, of knowledge of, attitudes towards, and practices relating to HIVST, following a programme introducing HIVST via targeted populations, allowing some spill-over effects to be captured. Secondly, unlike some previous studies, it assessed the levels of HIVST knowledge, attitudes and use among men and women separately, as well as their correlates, enabling more detailed policy recommendations.

The study suggests, for example, that actions aimed at improving general HIV-related knowledge and reducing stigma could help improve attitudes toward HIVST. Additionally, improving access to the media and providing more information about HIVST through these channels could enhance awareness. Less educated and poorer groups have the lowest levels of KAP relating to HIVST and should therefore be the focus of policymakers. The high level of positive attitudes towards HIVST calls for a scaling-up of access to HIVST in the region through dedicated programmes or through its availability in private pharmacies at low prices. Working along these lines could help policymakers improve HIV testing and progress towards UNAIDS targets of achieving 95 % of PLHIV knowing their status by 2025.

# Acknowledgments

This analysis was supported by Unitaid (grant number 2018–23 ATLAS) through a collaborative agreement with Solthis. The authors would like to thank all participants in this study, all the people involved in the data collection process, and the field workers in Côte d’Ivoire. We would also like to thank the members of the ATLAS Team.

# Author contributions

ASF and JL conceived and designed the analysis. ASF wrote the first draft of the manuscript. ASF supervised the data collection for the ATLAS team. ASF, CK, and JL performed the statistical analysis. AV implemented the ATLAS programme operations. MCB, RS, AV, and JL participated in data interpretation, and provided critical feedback on the manuscript. All the authors read and approved the final manuscript.

# References

1.	UNAIDS. The Urgency of Now: AIDS at a Crossroads [Internet]. Geneva: UNAIDS; 2024 [cited 2024 Aug 12] p. 300. Report No.: UNAIDS/JC3116E. Available from: https://www.unaids.org/en/resources/documents/2024/global-aids-update-2024

2.	WHO. Consolidated guidelines on HIV testing services 2019 [Internet]. Switzerland: WHO; 2020 [cited 2021 Feb 15] p. 292. Report No.: ISBN 978-92-4-155058-1. Available from: https://www.who.int/publications-detail-redirect/978-92-4-155058-1

3.	Simo Fotso A, Wright CG, Low A. How does HIV-related stigma correlate with HIV prevalence in African countries? Distinct perspectives from individuals living with and living without HIV. BMC Public Health. 2023 Sep 4;23(1):1720.

4.	Indravudh PP, Sibanda EL, d’Elbée M, Kumwenda MK, Ringwald B, Maringwa G, et al. “I will choose when to test, where I want to test”: investigating young people’s preferences for HIV self-testing in Malawi and Zimbabwe. AIDS Lond Engl. 2017 Jul 1;31 Suppl 3(Suppl 3):S203–12.

5.	Knight L, Makusha T, Lim J, Peck R, Taegtmeyer M, van Rooyen H. “I think it is right”: a qualitative exploration of the acceptability and desired future use of oral swab and finger-prick HIV self-tests by lay users in KwaZulu-Natal, South Africa. BMC Res Notes. 2017 Dec;10(1).

6.	Pai NP, Behlim T, Abrahams L, Vadnais C, Shivkumar S, Pillay S, et al. Will an Unsupervised Self-Testing Strategy for HIV Work in Health Care Workers of South Africa? A Cross Sectional Pilot Feasibility Study. PLOS ONE. 2013 Nov 27;8(11):e79772.

7.	Indravudh PP, Choko AT, Corbett EL. Scaling up HIV self-testing in sub-Saharan Africa: a review of technology, policy and evidence. Curr Opin Infect Dis. 2018 Feb;31(1):14.

8.	Choko AT, Kumwenda MK, Johnson CC, Sakala DW, Chikalipo MC, Fielding K, et al. Acceptability of woman-delivered HIV self-testing to the male partner, and additional interventions: a qualitative study of antenatal care participants in Malawi. J Int AIDS Soc. 2017;20(1):21610.

9.	Hector J, Davies MA, Dekker-Boersema J, Aly MM, Abdalad CCA, Langa EBR, et al. Acceptability and performance of a directly assisted oral HIV self-testing intervention in adolescents in rural Mozambique. Yotebieng M, editor. PLOS ONE. 2018 Apr 5;13(4):e0195391.

10.	Harichund C, Moshabela M. Acceptability of HIV Self-Testing in Sub-Saharan Africa: Scoping Study. AIDS Behav. 2018 Feb;22(2):560–8.

11.	Kra AK, Fotso AS, N’guessan KN, Geoffroy O, Younoussa S, Kabemba OK, et al. Can HIV self-testing reach first-time testers? A telephone survey among self-test end users in Côte d’Ivoire, Mali, and Senegal. BMC Infect Dis. 2023 Sep 25;22(1):972.

12.	Ky-Zerbo O, Desclaux A, Boye S, Maheu-Giroux M, Rouveau N, Vautier A, et al. “I take it and give it to my partners who will give it to their partners”: Secondary distribution of HIV self-tests by key populations in Côte d’Ivoire, Mali, and Senegal. BMC Infect Dis. 2023 May 24;22(1):970.

13.	Kra AK, Fotso AS, Rouveau N, Maheu-Giroux M, Boily MC, Silhol R, et al. HIV self-testing positivity rate and linkage to confirmatory testing and care: a telephone survey in Côte d’Ivoire, Mali, and Senegal. Peer Community J. 2024 Jun 13;4:e56.

14.	Chanda MM, Ortblad KF, Mwale M, Chongo S, Kanchele C, Kamungoma N, et al. HIV self-testing among female sex workers in Zambia: A cluster randomized controlled trial. PLoS Med. 2017 Nov;14(11):e1002442.

15.	Figueroa C, Johnson C, Verster A, Baggaley R. Attitudes and Acceptability on HIV Self-testing Among Key Populations: A Literature Review. AIDS Behav. 2015 Nov;19(11):1949–65.

16.	Ky-Zerbo O, Desclaux A, Boye S, Vautier A, Rouveau N, Kouadio BA, et al. Willingness to use and distribute HIV self-test kits to clients and partners: A qualitative analysis of female sex workers’ collective opinion and attitude in Côte d’Ivoire, Mali, and Senegal. Womens Health Lond Engl. 2022 Dec;18:17455057221092268.

17.	Grésenguet G, Longo J de D, Tonen-Wolyec S, Bouassa RSM, Belec L. Acceptability and Usability Evaluation of Finger-Stick Whole Blood HIV Self-Test as An HIV Screening Tool Adapted to The General Public in The Central African Republic. Open AIDS J. 2017 Nov 21;11(1):101–18.

18.	Simo Fotso A, Johnson C, Vautier A, Kouamé KB, Diop PM, Silhol R, et al. Routine programmatic data show a positive population-level impact of HIV self-testing: the case of Côte d’Ivoire and implications for implementation. AIDS. 2022;10.1097/QAD.0000000000003328.

19.	Silhol R, Maheu-Giroux M, Soni N, Simo Fotso A, Rouveau N, Vautier A, et al. Potential population-level effects of HIV self-test distribution among key populations in Côte d’Ivoire, Mali, and Senegal: a mathematical modelling analysis. Lancet HIV. 2024 Aug 1;11(8):e531–41.

20.	STAR project [Internet]. Available from: http://hivstar.lshtm.ac.uk/

21.	Rouveau N, Ky-Zerbo O, Boye S, Simo Fotso A, d’Elbée M, Maheu-Giroux M, et al. Describing, analysing and understanding the effects of the introduction of HIV self-testing in West Africa through the ATLAS programme in Côte d’Ivoire, Mali and Senegal. BMC Public Health. 2021 Jan 21;21(1):181.

22.	AIDSinfo | UNAIDS [Internet]. [cited 2024 Aug 12]. Available from: https://aidsinfo.unaids.org/

23.	Terefe B, Jembere MM, Reda GB, Asgedom DK, Assefa SK, Lakew AM. Knowledge, and utilization of HIV self-testing, and its associated factors among women in sub–Saharan Africa: evidence from 21 countries demographic and health survey. BMC Public Health. 2024 Jul 23;24(1):1960.

24.	Eholié S, Larmarange J, Ben Mechlia M, Mercier N, Nouaman MN, Coffie P, et al. ANRS 12381 PRINCESSE : PrEP, infections sexuellement transmissibles, contraception, hépatite virale B, santé sexuelle pour les travailleuses du sexe en Côte d’Ivoire [Internet]. ANRS ; Programme PAC-CI ; Institut de Recherche pour le Développement (IRD); 2022 Apr [cited 2024 Sep 19]. Available from: https://hal.science/hal-04127364

25.	Fox J, Monette G. Generalized Collinearity Diagnostics. J Am Stat Assoc. 1992 Mar 1;87(417):178–83.

26.	Nahhas RW. 5.20 Collinearity | Introduction to Regression Methods for Public Health Using R [Internet]. [cited 2024 Aug 16]. Available from: https://www.bookdown.org/rwnahhas/RMPH/mlr-collinearity.html

27.	James G, Witten D, Hastie T, Tibshirani R. An Introduction to Statistical Learning with Applications in R [Internet]. First. New York: Springer; 2013 [cited 2024 Aug 16]. 441 p. (Springer Texts in Statistics). Available from: https://www.statlearning.com

28.	Olakunde BO, Alemu D, Conserve DF, Mathai M, Mak’anyengo MO, NAHEDO Study Group, et al. Awareness of and willingness to use oral HIV self-test kits among Kenyan young adults living in informal urban settlements: a cross-sectional survey. AIDS Care. 2023 Sep;35(9):1259–69.

29.	Dzinamarira T, Muvunyi CM, Kamanzi C, Mashamba-Thompson TP. HIV self-testing in Rwanda: awareness and acceptability among male clinic attendees in Kigali, Rwanda: A cross-sectional survey. Heliyon. 2020 Mar 1;6(3):e03515.

30.	Wanga V, Omollo V, Bukusi EA, Odoyo JB, Morton JF, Kidoguchi L, et al. Uptake and impact of facility-based HIV self-testing on PrEP delivery: a pilot study among young women in Kisumu, Kenya. J Int AIDS Soc. 2020 Aug;23(8):e25561.

31.	Shava E, Manyake K, Mdluli C, Maribe K, Monnapula N, Nkomo B, et al. Acceptability of oral HIV self-testing among female sex workers in Gaborone, Botswana. Torpey K, editor. PLOS ONE. 2020 Jul 27;15(7):e0236052.

32.	Wachinger J, Kibuuka Musoke D, Oldenburg CE, Bärnighausen T, Ortblad KF, McMahon SA. “But I Gathered My Courage”: HIV Self-Testing as a Pathway of Empowerment Among Ugandan Female Sex Workers. Qual Health Res. 2021 Feb;31(3):443–57.

33.	Burke VM, Nakyanjo N, Ddaaki W, Payne C, Hutchinson N, Wawer MJ, et al. HIV self-testing values and preferences among sex workers, fishermen, and mainland community members in Rakai, Uganda: A qualitative study. Nikolopoulos GΚ, editor. PLOS ONE. 2017 Aug 16;12(8):e0183280.

34.	Ekholuenetale M, Nzoputam CI, Okonji OC. Association between socio-economic factors and HIV self-testing knowledge amongst South African women. South Afr J HIV Med. 2022 Mar 24;23(1):1347.

35.	Nibret Eskezia B, Tafere Y, Aschale A, Abebe Moges N. Uptake of HIV Self-Testing and Associated Factors Among Female Sex Workers at Non-Governmental HIV Testing Facilities in Debre Markos and Bahir Dar Towns, Northwest Ethiopia, 2022. HIVAIDS Auckl NZ. 2023;15:279–91.

36.	Flowers P, Riddell J, Park C, Ahmed B, Young I, Frankis J, et al. Preparedness for use of the rapid result HIV self-test by gay men and other men who have sex with men (MSM): a mixed methods exploratory study among MSM and those involved in HIV prevention and care. HIV Med. 2017 Apr;18(4):245–55.

37.	Mokgatle MM, Madiba S. High Acceptability of HIV Self-Testing among Technical Vocational Education and Training College Students in Gauteng and North West Province: What Are the Implications for the Scale Up in South Africa? PLOS ONE. 2017 Jan 31;12(1):e0169765.

38.	Sullivan MC, Rosen AO, Allen A, Benbella D, Camacho G, Cortopassi AC, et al. Falling Short of the First 90: HIV Stigma and HIV Testing Research in the 90–90–90 Era. AIDS Behav. 2020 Feb 1;24(2):357–62.

39.	Shafik N, Deeb S, Srithanaviboonchai K, Ayood P, Malasao R, Siviroj P, et al. Awareness and Attitudes Toward HIV Self-Testing in Northern Thailand. Int J Environ Res Public Health. 2021 Feb;18(3):852.

40.	Simo Fotso A, Kra AK, Maheu-Giroux M, Boye S, d’Elbée M, Ky-zerbo O, et al. Is it possible to recruit HIV self-test users for an anonymous phone-based survey using passive recruitment without financial incentives? Lessons learned from a pilot study in Côte d’Ivoire. Pilot Feasibility Stud [Internet]. 2022 [cited 2022 Jan 7];8(1). Available from: https://link.springer.com/epdf/10.1186/s40814-021-00965-2

# Supporting information

Table A1: Odds ratios from the multivariable logistic regression for KAP relating to HIVST among men aged 15-49 years in Côte d’Ivoire

Table A2: Odds ratios from the multivariable logistic regression for KAP relating to HIVST among women aged 15-49 years in Côte d’Ivoire

Supplements 1: Questions about HIV knowledge used to construct the knowledge score

Supplement 2: HIV-related stigma questions used to construct the stigma score
