# Supplementary material for: Knowledge, attitudes and practices relating to HIV self-testing following its introduction in the Bas-Sassandra region of Côte d’Ivoire: the case of the ATLAS project
Source: PLoS One. 2026 Jan 29;21(1):e0314947. doi: 10.1371/journal.pone.0314947 (PMC12854474; doi:10.1371/journal.pone.0314947)
Supplement: Attachment — Submitted filename: Response to Reviewers.docx [file pone.0314947.s009.docx]

Response to Reviewers and Editor

*We thank the Editor and reviewers for their helpful comments, which have improved the manuscript. Below, you will find our responses in purple to each point raised by the academic editor and reviewers.*

# Editor Comments

Journal Requirements:

When submitting your revision, we need you to address these additional requirements.

1. Please ensure that your manuscript meets PLOS ONE's style requirements, including those for file naming. The PLOS ONE style templates can be found at

<https://journals.plos.org/plosone/s/file?id=wjVg/PLOSOne_formatting_sample_main_body.pdf> and 
<https://journals.plos.org/plosone/s/file?id=ba62/PLOSOne_formatting_sample_title_authors_affiliations.pdf>

*The manuscript style has been updated accordingly*

2. Please include a complete copy of PLOS’ questionnaire on inclusivity in global research in your revised manuscript. Our policy for research in this area aims to improve transparency in the reporting of research performed outside of researchers’ own country or community. The policy applies to researchers who have travelled to a different country to conduct research, research with Indigenous populations or their lands, and research on cultural artefacts. The questionnaire can also be requested at the journal’s discretion for any other submissions, even if these conditions are not met.

Please find more information on the policy and a link to download a blank copy of the questionnaire here: <https://journals.plos.org/plosone/s/best-practices-in-research-reporting>.

Please upload a completed version of your questionnaire as Supporting Information when you resubmit your manuscript.

*The questionnaire has been uploaded.*

3. Thank you for stating the following financial disclosure:

“This analysis was supported by Unitaid (grant number 2018–23 ATLAS) through a collaborative agreement with Solthis.”

Please state what role the funders took in the study.  If the funders had no role, please state: "The funders had no role in study design, data collection and analysis, decision to publish, or preparation of the manuscript." If this statement is not correct you must amend it as needed.

Please include this amended Role of Funder statement in your cover letter; we will change the online submission form on your behalf.

*The cover letter has been amended with the following statement “This analysis was supported by Unitaid (grant number 2018–23 ATLAS) through a collaborative agreement with Solthis. The funders had no role in study design, data collection and analysis, decision to publish, or preparation of the manuscript.”*

4. Please note that funding information should not appear in the Acknowledgments section or other areas of your manuscript. We will only publish funding information present in the Funding Statement section of the online submission form. Please remove any funding-related text from the manuscript.

*The funding-related information has been removed from the acknowledgements as suggested*

5. We note that you have indicated that there are restrictions to data sharing for this study. For studies involving human research participant data or other sensitive data, we encourage authors to share de-identified or anonymized data. However, when data cannot be publicly shared for ethical reasons, we allow authors to make their data sets available upon request. For information on unacceptable data access restrictions, please see <http://journals.plos.org/plosone/s/data-availability#loc-unacceptable-data-access-restrictions>.

Before we proceed with your manuscript, please address the following prompts:

a) If there are ethical or legal restrictions on sharing a de-identified data set, please explain them in detail (e.g., data contain potentially identifying or sensitive patient information, data are owned by a third-party organization, etc.) and who has imposed them (e.g., a Research Ethics Committee or Institutional Review Board, etc.). Please also provide contact information for a data access committee, ethics committee, or other institutional body to which data requests may be sent.

b) If there are no restrictions, please upload the minimal anonymized data set necessary to replicate your study findings to a stable, public repository and provide us with the relevant URLs, DOIs, or accession numbers. Please see <http://www.bmj.com/content/340/bmj.c181.long> for guidelines on how to de-identify and prepare clinical data for publication. For a list of recommended repositories, please see <https://journals.plos.org/plosone/s/recommended-repositories>. You also have the option of uploading the data as Supporting Information files, but we would recommend depositing data directly to a data repository if possible.

Please update your Data Availability statement in the submission form accordingly.

*The minimal anonymized dataset and R script used to replicate the presented analyses have been made publicly available on Zenodo with the DOI: 10.5281/zenodo.15064708 .*

*The full survey data are considered only pseudonymized according to the European General Data Protection Regulation (GDPR) and therefore cannot be made publicly available. They can only be shared under specific restrictions by contacting the corresponding authors.*

*Data Availability statement has been updated accordingly.*

6. In the online submission form, you indicated that:

“Data cannot be shared publicly at this time, as it was collected through a joint survey conducted by the ATLAS program and the MTV Shuga initiative. As members of the ATLAS program team, we can only share the data once MTV Shuga has published results on the primary outcomes of their project. In the meantime, the data will be available upon request, and we have included it as a supplementary document during the submission process for reviewers’ reference. Ultimately, the data will be made publicly available on Zenodo.”

All PLOS journals now require all data underlying the findings described in their manuscript to be freely available to other researchers, either 1. In a public repository, 2. Within the manuscript itself, or 3. Uploaded as supplementary information.

This policy applies to all data except where public deposition would breach compliance with the protocol approved by your research ethics board. If your data cannot be made publicly available for ethical or legal reasons (e.g., public availability would compromise patient privacy), please explain your reasons on resubmission and your exemption request will be escalated for approval.

*The minimal anonymized dataset and R script used to replicate the presented analyses have been made publicly available on Zenodo with the DOI: 10.5281/zenodo.15064708 . See response to comment 6*

7. One of the noted authors is a group or consortium: ATLAS team

In addition to naming the author group, please list the individual authors and affiliations within this group in the acknowledgments section of your manuscript. Please also indicate clearly a lead author for this group along with a contact email address.

*Individuals from the ATLAS group have been named in the acknowledgments. Note that they do not meet the criteria for authorship of this paper; that is why we used “on behalf of the…” instead of “and the…” in the author list.*

8. We are unable to open your Supporting Information file data.zip. Please kindly revise as necessary and re-upload.

*The minimal anonymized dataset and R script used to replicate the presented analyses have been made publicly available on Zenodo with the DOI: 10.5281/zenodo.15064708 . See response to comments 6 and 7*

9. Please review your reference list to ensure that it is complete and correct. If you have cited papers that have been retracted, please include the rationale for doing so in the manuscript text, or remove these references and replace them with relevant current references. Any changes to the reference list should be mentioned in the rebuttal letter that accompanies your revised manuscript. If you need to cite a retracted article, indicate the article’s retracted status in the References list and also include a citation and full reference for the retraction notice.

*Our reference list is complete and correct.*

# Additional Editor Comments: Investigating knowledge about HIV self-testing is crucial for strengthening public health strategies to increase testing rates and reduce HIV-related stigma. While our study provides significant evidence in this area, there are several key issues that still need to be addressed. 1- Include keywords derived from MeSH terms.

*The following keywords derived from MeSH were included: Health Knowledge, Attitudes, Practice; Self-Testing; HIV Infections; Humans, Cote d’Ivoire; Cross-Sectional Studies*

2- The introduction does not transition smoothly from general concepts to specific details. Please revise the introduction to ensure clarity and maintain coherence.

*The introduction was revised accordingly*

3- Please include the sample size formula as well as the criteria for inclusion and exclusion in the study.

*The following paragraph was added to the method section:*

*“**The sample size was calculated to ensure that the study's power was estimated at 92% and 99% to detect a minimum difference of 10% and 15%, respectively, in the ATLAS primary outcome compared to the 2018 PHIA (Population-Based HIV Impact Assessment) survey. The inclusion criteria were individuals aged 15 to 49 years, de facto household members (i.e., those present in the household at the time of the survey), individuals aged 18 or older with written informed consent, and those aged 15–17 with written informed assent and parent/guardian's written informed consent. The exclusion criteria included individuals younger than 15 or older than 49 years, those not present in the household at the time of the survey, refusal by the participant and/or parent/guardian, refusal by individuals aged 18 or older, refusal by a parent/guardian or lack of assent from individuals aged 15–17, and cognitive issues preventing the individual from providing informed consent.”*

4- Please provide the content of the knowledge, attitude, and practice survey form, including the number of questions and the scoring method used.

*The questions about HIV self-testing (HIVST) knowledge, attitude, and practice are now provided in Supporting Information S1 Table. No special scoring method was used, as these were binary variables. To clarify how these variables were coded, the manuscript has been amended as follows:*

*“We defined four dichotomous dependent variables related to knowledge, attitudes, and practices relating to HIV self-testing derived from the questions presented in the supporting information S1 Table. The first dependent variable measured individuals’ knowledge of HIVST, assessing whether the respondent had already heard of HIV self-testing, coded as “yes” or “no”. The second and third dependent variables concerned the respondents’ interest in using HIVST for themselves or for their partners if it was freely available. Each of these were binary variables indicating whether the individuals responded that they interested /very interested or not. These variables reflected the respondents’ attitudes towards HIVST. The fourth variable reflecting practices asked individuals whether they had ever used HIVST. This was also a binary variable, coded as “yes” or “no”.”*

5- In the methods section on page 6, it is unclear whether HIV-related knowledge and stigma or attitude have been investigated. Please clarify, as attitude and stigma are distinct concepts and should be addressed separately.

*In the literature, HIV-related stigma is often seen as an umbrella concept and "refers to beliefs and/or attitudes about HIV" (see the articles doi: 10.1002/jia2.25915 or doi: 10.1007/s10461-009-9593-3). However, to address the reviewer's suggestion and given that our specific questions are mainly related to attitudes towards PLHIV, we have changed the wording to "negative attitudes toward PLHIV.*

6- Please include the Ethics Committee Code in the manuscript.

*Ethics committee codes have been included in the manuscript page 6 as follow “**Ethics approvals were obtained from the Côte d’Ivoire Ministry of Health (N/ref: 051-21/MSHP/CNESVS-km), the London School of Hygiene and Tropical Medicine (LSHTM) (LSHTM Ethics Ref: 26258), and the World Health Organization (WHO) (ERC.0003596) ethics committees in June, July and September 2021 respectively.” It was also added to acknowledgement as per editor’s suggestion*

# Reviewer #1:

This manusccrips describes apect knowlledge attitude and practices towrads HIV self testing the Bas-Sassandra region of Côte d’Ivoire. The reserach questions were carefully enunciateds, the design and sampling methods were sound and the analystical techniques employed were appropriate considering the design and research questions.

Overall the manuscript was well written and it was joy to read. The authors report on some signoificant findings which although not novell does provide the foundation to faciliatate evaluation of programme effficay should scaling be pursued

*We thank the reviewer for their very positive comments regarding the paper.*

# Reviewer #2:

1. Keyword based on mesh is not

*Comment addressed. Please see our response to editor’s additional comment number 1.*

2. The introduction does not proceed clearly from generalities to details.

*Comment addressed. Please see our response to editor’s comment number 2.*

3. Sample size formula?

*Comment addressed. Please see our response to editor’s additional comment number 3.*

4. Study inclusion and exclusion criteria?

*Comment addressed. Please see our response to editor’s additional comment number 3.*

5. Content of the knowledge, attitude, and practice survey form (number of questions) and its scoring method.

*Comment addressed. Please see our response to editor’s additional comment number 4.*

6. Is it attitude or stigma that has been investigated?

*Comment addressed. Please see our response to editor’s additional comment number 5.*

7. Attitude and stigma are two separate concepts.

*Comment addressed. Please see our response to editor’s additional comment number 6.*

8. Ethics Committee Code?

*Comment addressed. Please see our response to editor’s additional comment number 7.*
