# Supplementary material for: Knowledge, attitudes and practices relating to HIV self-testing following its introduction in the Bas-Sassandra region of Côte d’Ivoire: the case of the ATLAS project
Source: PLoS One. 2026 Jan 29;21(1):e0314947. doi: 10.1371/journal.pone.0314947 (PMC12854474; doi:10.1371/journal.pone.0314947)
Supplement: Attachment — Submitted filename: Response to reviewers comment.docx [file pone.0314947.s010.docx]

**Response to reviewers’ comment.**

We thank the reviewers and the editor for their very positive comments.

Only point 6 required a response from us: However one area that needs addressing, is the abstract - there is no indication of the number of people interviewed, where they came from, or how they were selected - a sentence or two on this would make all the difference.

We have addressed this comment by adding the following sentences to the abstract: “A total of 6,271 people (3,203 men and 3,068 women) were interviewed. They were selected using a three-stage stratified sampling approach in the Bas-Sassandra region.”
